# Household and community HIV/AIDS status and child malnutrition in sub-Saharan Africa: Evidence from the demographic and health surveys

**DOI:** 10.1016/j.socscimed.2011.05.042

**Published:** 2011-08

**Authors:** Monica A. Magadi

**Affiliations:** Department of Sociology, City University London, London EC1V 0HB, UK

**Keywords:** Sub-saharan Africa, Child malnutrition, HIV/AIDS risk factors, Orphaned children, Vulnerable children, Demographic and health surveys, Contextual factors

## Abstract

This paper examines the extent to which under five children in households or communities adversely affected by HIV/AIDS are disadvantaged, in comparison with other children in less affected households/communities. The study is based on secondary analysis of the Demographic and Health Survey (DHS) data collected during 2003–2008 from 18 countries in sub-Saharan Africa, where the DHS has included HIV test data for adults of reproductive age. We apply multilevel logistic regression models that take into account the effect of contextual community/country level HIV/AIDS factors on child malnutrition. The outcome variable of interest is child undernutrition: stunting, wasting and underweight. The results suggest that across countries in sub-Saharan Africa, children whose mothers are infected with HIV are significantly more likely to be stunted, wasted or underweight compared to their counterparts of similar demographic and socio-economic background whose mothers are not infected. However, the nutritional status of children who are paternal orphans or in households where other adults are HIV positive are not significantly different from non-orphaned children or those in households where no adult is infected with HIV. Other adult household members being HIV positive is, however, associated with higher malnutrition among younger children below the age of one. Further analysis reveals that the effect of mothers’ HIV status on child nutritional status (underweight) varies significantly across communities within countries, the effect being lower in communities with generally higher levels of malnutrition. Overall, the findings have important implications for policy and programme efforts towards improved integration of HIV/AIDS and child nutrition services in affected communities and other sub-groups of the population made vulnerable by HIV/AIDS. In particular, children whose mothers are infected with HIV deserve special attention.

## Introduction

### Background

Sub-Saharan Africa remains the region most adversely affected by the HIV/AIDS epidemic, accounting for 68 percent of the global burden in 2009, and despite recent declines in new infections, the number of people living with HIV/AIDS has continued to grow (UNAIDS, 2010). The HIV/AIDS epidemic in sub-Saharan Africa is believed to have retarded progress in the reduction of under five mortality in the region through its impoverishing effects which impinge on the health and nutrition of children, or through illness of the parent and/or the child. Almost all countries in sub-Saharan Africa with severe HIV/AIDS epidemics have made insufficient or no progress towards meeting the Millennium Development Goal (MDG) 4 on child mortality reduction (UNICEF, WHO, The World Bank, & the United Nations Population Division, 2010). Furthermore, sub-Saharan Africa has made little or no progress towards the World Food Summit or the Millennium Development Goals to halve the number of people undernourished between 1990 and 2015 (FAO, 2009). The WHO consultation on nutrition and HIV/AIDS in Africa recognized that ‘far-reaching steps need to be taken to reverse current trends in malnutrition, HIV infection and food insecurity in most countries in the region, in order to achieve the Millennium Development Goals’ (WHO, 2005:3). An improved understanding of how HIV/AIDS affects child malnutrition in the worst affected regions will help inform such efforts.

Malnutrition and HIV/AIDS are intertwined in a vicious circle: whilst HIV infection heightens vulnerability to malnutrition, malnutrition on the other hand degrades the immune system and heightens vulnerability to HIV transmission risk and disease progression (Anema, Vogenthaler, Frongillo, Kadiyala, & Weiser, 2009; Saloojee, De Maayer, Garenne, & Kahn, 2007). In this paper, we focus on the possible effect of HIV/AIDS epidemic on nutritional status of children in sub-Saharan Africa.

HIV/AIDS can affect the health and nutritional status of children in a number of ways. Children may be affected by HIV/AIDS indirectly or directly when their communities, and the services these communities provide, are strained by the consequences of the AIDS epidemic or when they become orphans, have ill parents, live in poor households that have taken in orphans, are discriminated against because of a family member’s HIV status, or have HIV themselves’ (UNICEF, 2006). Firstly, HIV/AIDS may affect children directly when the children themselves get infected with HIV from vertical transmission. Existing studies suggest that children who are HIV-infected are more likely to suffer malnutrition (Bunn, 2009; Nalwoga et al., 2010) and their survival is also at jeopardy (Fergusson & Tomkins, 2009; Fergusson, Tomkins, & Kerac, 2009). In a study of nutritional status of children living in a community with high HIV prevalence in rural Uganda, Nalwoga et al. (2010) observed that the prevalence of undernutrition (underweight and stunting) was significantly higher in HIV positive than in HIV-negative children. This is consistent with earlier findings from other settings in sub-Saharan Africa (Bunn, 2009).

Secondly, HIV/AIDS may affect children as a consequence of parental illness and death. It has been noted that children born to HIV positive women are more likely to die before the age of five than other children, and this risk applies to all these children and not just those who are HIV-infected themselves (Nakiyingi et al., 2003). However, empirical evidence on the effect of HIV status of adult household members on nutritional status of children remains inconclusive. While there is evidence that maternal survival and HIV status are strong predictors of infant and child survival (Nakiyingi et al., 2003), some studies suggest that there is no significant difference in the prevalence of indicators of undernutrition in children classified by maternal HIV and survival status or where a family member is sick with AIDS (Bridge, Kipp, Jhangri, Laing, & Konde-Lule, 2006; Nalwoga et al., 2010). Furthermore, many studies have shown that orphans do not usually have poorer health than non-orphans in the same community. Although modest increases in ill-health and malnutrition were found in orphans in the Demographic and Health Surveys data, with maternal and double orphans being worst affected (Owen et al, 2009), no association between orphanhood and nutritional status have been found in various settings in sub-Saharan Africa (Owen et al., 2009; Zidron, Juma, & Ice, 2009).

Furthermore, HIV/AIDS can affect children through the impoverishing effects of HIV/AIDS. Worsening poverty has been identified as one of the impacts of HIV/AIDS on children, their families and communities, affecting the health and nutrition of children in a number of ways. In particular, the nutritional status of a child in an AIDS-affected household might be impacted through reduced household agricultural and economic productivity, leading to household food insecurity including food insufficiency. Food insufficiency may in turn lead to childhood malnutrition (stunting, wasting, underweight) due to social and biological vulnerabilities of children. A survey of the effect of prime-age mortality (largely from HIV/AIDS) on crop production, cropping patterns, and nonfarm income in rural farm households of Zambia revealed that prime-age mortality severely affected the farm production (Chapoto & Jayne, 2008).

Overall, HIV/AIDS may have negative effects on the growth and development of children, and causes of this could include poverty and illness of the parent and/or the child. However, evidence from existing studies conducted in various settings in sub-Saharan Africa have produced different results and remain inconclusive. For instance, some have shown that orphans are more likely to be underweight, wasted or stunted than non-orphans, while other studies have failed to show any significant difference (Owen et al., 2009; Zidron et al., 2009). The lack of conclusive patterns from previous studies is mainly attributable to the fact that most of these studies have focused on specific settings, and sometimes involving small samples with limited statistical power to detect significant associations. This study aims to contribute to current knowledge on the effect of household/community HIV/AIDS status on child malnutrition in sub-Saharan Africa. It involves a comprehensive analysis of pooled data from different countries in sub-Saharan Africa to provide an overall picture for the region, useful for informing international efforts to address the HIV/AIDS crisis and its adverse impact on child health.

### Aims and objectives

In this paper, we carry out a cross-sectional analysis of the family/household and community HIV/AIDS determinants of infant and child health across countries in sub-Saharan Africa. We focus on malnutrition as an important indicator of child health, and examine the extent to which children whose mothers are infected with HIV, or in households or communities adversely affected by HIV/AIDS are disadvantaged, in comparison with other children in less affected households or communities. The specific objectives are to:

(i) examine the effect of survival and HIV status of parents and other adult household members on nutritional status of children aged under five in sub-Saharan Africa;
(ii) establish the effect of contextual community or national HIV prevalence on child malnutrition; and
(iii) explore community and national variations in the association between survival/HIV status of household members and nutritional status of children

An examination of the effect of orphanhood and HIV/AIDS status of family members on child malnutrition is necessary to identify the most vulnerable children and families in order to inform efforts aimed at addressing the adverse impact of HIV/AIDS on children. Besides family/household HIV/AIDS effects on child malnutrition, the paper places particular emphasis on country and community variations in the association between HIV/AIDS and nutritional status of children, and the extent of clustering of malnutrition within countries and communities (clusters within country). The degree to which child malnutrition is clustered within particular areas has important implications for effectiveness of geographic targeting in nutrition programmes (Fenn, Morris, & Frost, 2004).

## Data and methods

### The data

The paper is based on secondary analysis of existing data from the international Demographic and Health Surveys (DHS) programme from different countries in sub-Saharan Africa (SSA). Since 1984, the DHS has collected nationally representative data on population, health, and nutrition from 85 developing countries. New topics have been added during the more recent period to address emerging health issues, including HIV/AIDS. The DHS ‘use consistent sampling methodologies and questions, ensuring comparability among countries and over time while still maintaining flexibility to meet individual country needs’ (ICF Macro, 2010b, p.4). This study uses recent DHS data collected from 18 countries in SSA during 2003–2008. The comparative nature of DHS data, along with the availability of HIV test data from recent surveys, provides a unique opportunity for a population-based study of factors associated with the HIV/AIDS epidemic in different contexts. The data analysed in this paper relate to children aged under five in households selected for HIV testing, the summary of which is given in Table 1. The DHS HIV testing protocol undergoes a rigorous ethical review process (ICF Macro, 2010a), providing for informed, anonymous, and voluntary testing of women and men of reproductive age.

### Methods of analysis

The outcome variable of interest is child undernutrition (stunting, wasting and underweight). Anthropometric indicators of nutritional status (height-for-age, weight-for-age and weight-for-height) were used to define nutritional status of children. Children with a Z score less than −2 relative to the World Health Organization (WHO) standards are defined as undernourished (WHO, 2006): weight-for-age defining underweight; height-for-age defining stunting; and weight-for-height defining wasting. Deviations below −2 standard deviations indicate that the children are moderately or severely undernourished. Each of these indicators measure a different aspect of the nutritional status of children: chronic undernutrition (stunting), acute undernutrition (wasting) and general undernutrition (underweight). Although malnutrition includes both under– and over-nutrition, this paper focuses on undernutrition which is a greater concern in the developing countries, contributing significantly to under five mortality (Mukuria, Cushing, & Sangha, 2005).

The key explanatory variables relate to survival and HIV status of parents and other adult household members (i.e. mother’s HIV status, HIV status of other adult household members, and paternal orphanhood status), as well as contextual HIV/AIDS factors at community and country level. Although maternal (or double) orphanhood is potentially an important factor in child’s nutritional status, it was not possible to include maternal orphanhood in the analysis since the analysis sample largely comprised children whose mothers were available (as respondents) to provide key information used in the analysis. Since the DHS data are limited on community level or country level information, all contextual factors considered in the analysis were derived from relevant individual-level data with the exception of country level GDP per capita^1^. Thus, contextual HIV data relating to community (cluster) and country level HIV prevalence, and the prevalence of orphanhood have been derived from individual-level information. Other explanatory variables expected to be associated with child nutritional status (Giroux, 2008; Mbuya, Chideme, Chasekwa, & Mishra, 2010; Mukuria et al., 2005) were included in the analysis as covariates (see Appendix i for a description of variables included in the analysis). These include:

- child-level characteristics (e.g age of child, sex of child, birth order, multiple/twin birth, preceding birth interval, breastfeeding experience, and size of child at birth);
- maternal characterstics (i.e mother’s age, educational attainment, marital status); and
- household factors and residence (household wealth index, urban/rural residence).

The analysis is based on multilevel modeling, taking into account the hierarchical data structure resulting from pooling data across countries and the DHS survey design (individuals nested within communities/clusters which are in turn nested within countries). We recognize the complex hierarchical data structure potentially comprising five different levels in the data: children within women within households within clusters within countries. Preliminary analysis assessed significance of all these levels. However, women and household levels were dropped since there was no evidence of significant variations in child malnutrition at these levels, presumably due to the relatively small number of children per woman or household.

The multilevel analysis also allows for inclusion of contextual HIV/AIDS factors at community and country levels in the model. The modeling features random coefficient models, allowing the effect of key explanatory variables relating to HIV status of adult household members and parental survival to vary across communities and countries. The general form of the multilevel logistic regression model used in the analysis may be expressed as:

(1) $${\text{Logit}\;\text{π}}_{ijk}=X_{ijk}^{\text{′}}\beta +Y_{ijk}^{\text{′}}u_{jk}+Z_{ijk}^{\text{′}}v_{k}$$

where: ${\text{π}}_{ijk}$ is the probability of being undernourished for a child *i*, in the $j^{th}$ community in the $k^{th}$ country; $X_{ijk}^{\text{′}}$ is the vector of covariates which may be defined at the individual/household, community or country level; β is the associated vector of usual regression parameter estimates; $Y_{ijk}^{\text{′}}$ is a vector of covariates (usually a subset of $X_{ijk}^{\text{′}}$) which vary randomly at community level; $Z_{ijk}^{\text{′}}$ is a vector of covariates (usually a subset of $X_{ijk}^{\text{′}}$) which vary randomly at country level; and the quantities $v_{k},\text{and}\;u_{jk}$ are the residuals at the country and community level, respectively. These are assumed to have normal distributions with mean zero and variances $\sigma_{v}^{2}\;\text{and}\;\sigma_{u}^{2}$ (Goldstein, 2003).

The estimates of country and community level variances are used to calculate intra-unit correlation coefficients to examine the extent to which the risk of child undernutrition is clustered within countries (or communities within countries) in sub-Saharan Africa. The degree of clustering is measured before and after taking into account the effect of significant covariates. Since children within the same community are also within the same country, the intra-community correlations include country variances (see, for example, Siddiqui, Hedeker, Flay, & Hu, 1996). Thus, the intra-community (*ρ_u_*) and intra-country (*ρ_v_*) correlation coefficients are given by:

(2) $$\rho_{u}=\frac{\sigma_{u}^{2}+\sigma_{v}^{2}}{\sigma_{v}^{2}+\sigma_{u}^{2}{+\text{π}}^{2}/3}$$

and

(3) $$\rho_{v}=\frac{\sigma_{v}^{2}}{\sigma_{v}^{2}+\sigma_{u}^{2}+{\text{π}}^{2}/3}$$

where: $\sigma_{v}^{2}$ – is the total variance at country level; $\sigma_{u}^{2}$ -is the total variance at community level; and ${\text{π}}^{2}/3$ – is the total variance at individual level.

For the multilevel logistic regression model, the level-1 residuals, $e_{ijk}$, are assumed to have a standard logistic distribution with mean zero and variance ${\text{π}}^{2}/3$, where π is the constant 3.1416 (see Hedeker & Gibbsons, 1996).

The issue of a sufficient sample size is an important consideration in multilevel analysis. Snijders (2005) points out that sample size determination in multilevel designs requires attention to the fact that statistical power depends on the total sample sizes for each level. Although a number of studies have addressed the issue of what constitutes a sufficient sample size in multilevel models, a consensus has yet to develop (Busing, 1993; Maas & Hox, 2005; Snijders & Bosker, 1999). For instance, while Busing (1993) recommends a minimum of 100 higher level units, Snijders and Bosker (1999, p44) state that multilevel modeling becomes attractive when the sample of groups is larger than 10. Simulation studies by Kreft (1996) based on two level models suggest an adequate statistical power with 30 groups of 30 observations each; 60 groups with 25 observations each; or 150 groups with 5 observations each, suggesting that a larger number of observations per group is required for a smaller number of groups. Moineddin, Matheson, and Glazier (2007) further noted that when group sizes are small (i.e. less than 5), convergence problems are likely to arise and random intercepts are severely overestimated. Although the number of Level-3 units in this paper (*n* = 18 countries) is relatively small, the large number of individuals per community and communities within country somewhat compensates for this. Overall, the average of eight cases per community/cluster across the 6808 clusters in 18 countries is fairly large, leading to relatively stable estimates and no convergence problems. It has been pointed out that power for individual-level estimates depends on the number of individuals, while power for higher level estimates depends on the number of groups (Kreft, 1996; Snijders, 2005). Thus, it is important to note that the relatively small number of countries in our analysis is likely to lead to reduced statistical power to detect significant country level effects, implying that only relatively large effect sizes at country level would be detected as significant. The analysis was undertaken using MLwiN multilevel software and estimations based on second order Predictive Quasi-Likelihood (PQL) procedure (Rasbash, Steele, Browne, & Prosser, 2005).

The analysis starts with an examination of the bivariate associations between survival/HIV status of adult household members and child undernutrition in each of the countries to understand the associations in specific countries, before controlling for the effect of important individual-level and contextual factors. The bivariate analyses are based on cross-tabulations with Chi–Square tests for significance. We recognize that the bivariate associations between nutritional status of children and parent’s survival and HIV status are likely to be affected by confounding factors, concealing or modifying the risk associated with vulnerability of children in households adversely affected by HIV/AIDS. For instance, previous studies have shown that those living in urban areas or in richer households are more likely to be infected with HIV (Magadi & Desta, 2009; Mishra et al., 2007). Since child malnutrition levels are likely to be lower among children in these same households, the above relationships are likely to be moderated by these factors. Therefore, the bivariate analyses are followed with multilevel logistic regression models, applied to pooled data across countries in sub-Saharan Africa to understand the general patterns across the region after taking into account important individual-level and contextual factors. For both bivariate and multivariate associations, a five percent cut-off point is used to determine statistical significance.

### Data limitations

We recognize important potential data limitations that should be borne in mind while interpreting our findings. The first relates to possible survivor bias of the sample of children included in the analysis. In cross-sectional surveys, the sample of children with anthropometric measurements is not representative of all children in a birth cohort, since only children surviving to the survey date are measured. Although this survivor bias could have implications for studies of trends and differentials in anthropometric indicators, comparisons of anthropometric data across geographic units, population sub-groups, and calendar time are only marginally affected by the survivor bias, unless mortality differences between the birth cohorts are very large (Boerma, Sommerfelt, & Bicego, 1992).

The second limitations relates to the data on parents’’survivorship status. The analysis has only included paternal survivorship, even though previous studies have suggested that it is maternal or double orphans who are more likely to suffer ill-health and malnutrition (Owen et al. 2009). It has not been possible to include maternal orphans since key information on the sample of children included in the analysis was mainly provided by the mothers (as respondents), implying that children whose mothers were not present were unlikely to be included in the analysis sample.

Thirdly, a number of potentially important contextual factors that may influence child malnutrition were missing and therefore not included in the analysis. Such factors may include income inequalities, local war context, violence, risky health behaviors (due to rituals, superstitions or taboos) that could be both at the origin of HIV seropositivity and of malnutrition. It is possible that these unobserved factors may moderate the observed relationships between HIV/AIDS and child malnutrition observed. Furthermore, limited contextual information implies that the full advantage of multilevel modeling could not be realized. Since the DHS data do not have contextual level information, all contextual variables considered in the analysis (except GDP per capita) were derived from individual level information.

Finally, it is important to recognize that although the countries included in the analysis (i.e DHS with HIV test data) provide good coverage of the diverse settings in sub-Saharan Africa, they do not necessarily constitute a random sample of all countries in the region. This limits the extent to which observed patterns may be generalized across all countries in sub-Saharan Africa.

## Results

In examining the association between household/community HIV/AIDS status and child malnutrition, it is important to first understand the bivariate distribution of child malnutrition by various HIV/AIDS characteristics to aid interpretation of the findings. Preliminary analysis examined the distribution of undernourished children in each country by basic demographic characteristics of children, namely: sex (Appendix ii) and age (Appendix iii a,b,c).

### Bivariate analysis

The association between HIV status of adult household members and stunting status of children aged under five shows mixed patterns (Table 2a). In countries where the relationship is significant, the highest proportion of children stunted are observed either among those whose mothers are infected with HIV (Ethiopia, Lesotho and Zimbabwe), or among those in household with no adult infected with HIV (DR Congo, Malawi or Zambia).

The bivariate relationship between HIV status of adult household members and wasting status of children under five is not significant in almost all countries (Table 2b). The only exception is Ghana and Guinea, and there is no clear pattern – the highest prevalence of wasting is among children in households where other adult members are HIV positive in Ghana, and among children whose mothers are HIV positive in Guinea.

The distribution of HIV status of adult household members by underweight status of children also shows mixed patterns (Table 2c). The highest proportion of children underweight is observed among those in households where no adult household member is HIV positive (Cameroon, DR Congo and Rwanda), or among children whose mothers are HIV positive (Kenya and Zimbabwe).

Overall, little significance is observed in the bivariate relationships between children’s nutritional status and paternal orphanhood status (Appendix iv a,b,c), and there is no clear evidence that paternal orphans are more disadvantaged compared to non-orphans. In countries where the relationship is significant, the prevalence of undernutrition is higher either among non-orphans (stunting in Sierra Leone; wasting in Mali), or among paternal orphans (wasting in Zambia and Zimbabwe; underweight in Zimbabwe).

In the next section, we examine the risk of undernutrition among children aged under five by HIV status of adult household members, paternal orphanhood status, and contextual HIV/AIDS factors, while controlling for the effect of various characteristics expected to be associated with child nutritional status and/or HIV infection.

### Multilevel multivariate analysis

The results of the multilevel logistic regression models showing the average odds ratios (and 95 percent confidence intervals) of factors associated with child undernutrition are presented in Table 3. The results suggest that across countries and communities in sub-Saharan Africa, children whose mothers are HIV positive are significantly more likely to be stunted, wasted or underweight than children in households where no adult is infected with HIV. On average, children whose mothers are HIV positive have a 26–28 percent higher odds of undernutrition than their counterparts of similar characteristics in households with no adult infected with HIV. The vulnerability of children whose mothers are HIV positive becomes particularly apparent once socio-economic factors (i.e. urban/rural residence, household wealth status, and mother’s educational attainment) are controlled for. Although there is no evidence that the overall risk of child undernutrition is higher among children aged under five in households where other adults are infected with HIV, an interaction of HIV status of adult household members (see Appendix v) suggests that the risk of undernutrition among children in households where adult members (or mother) are infected with HIV is escalated among younger children aged under one in relation to older children.

Unlike maternal HIV seropositivity which is associated with an increased risk of child undernutrition, paternal orphanhood is generally associated with a reduced risk. It is interesting to note that after controlling for important background characteristics, children who are paternal orphans are generally less likely to be stunted or underweight than their counterparts of similar characteristics who are not paternal orphans.

The risk of child undernutrition associated with the other covariates included in the model largely conform to what might be expected. The child characteristics with respect to age, sex, birth order, multiple/twin birth, preceding birth interval, breastfeeding duration are all significantly associated with undernutrition. The risk of stunting and underweight is generally higher among older children aged over one, male children, higher order births, shorter preceding birth interval less than two years, those breastfed for a longer duration, or who were small at birth. The pattern for wasting tend to follow a similar pattern for most of the indicators (except child’s age), but the associations tend to be weaker. Unlike stunting and underweight where the lowest risk is observed among the youngest children aged under one, the risk of wasting is lowest among the older children aged four.

The risk of child undernutrition by mother’s or household characteristics are generally consistent across the three measures of undernutrition, with children of teenage mothers, or whose mothers have no education, or in the poorest or single parent households having the highest risk of undernutrition. However, the risk of wasting is not significant by mothers’ age or single parenthood. Also, although rural residence is associated with increased risk of stunting and underweight, it is associated with reduced risk of wasting.

Although higher HIV prevalence in communities might be expected to be associated with a higher risk of undernutrition due to the impoverishing effects of HIV/AIDS in communities, it is interesting to note that the risk of stunting is in fact generally lower in communities with higher HIV prevalence. Although the risk of stunting and underweight also tended to be lower in countries of higher HIV prevalence, this relationship ceases to be significant when country level GPD per capita (which has a negative association with levels of stunting and underweight) is controlled for. However, it is interesting to note that the risk of wasting is lower in countries of higher HIV prevalence, even after country level GDP per capita is controlled for.

The results presented in Table 3 suggest that the risk of child undernutrition varies significantly across communities, and to a lesser extent across countries in sub-Saharan Africa. The variation in the level of child undernutrition across countries is partly explained by GDP per capita – inclusion of contextual factor relating to country-level GDP per capita in the model leads to a notable reduction in the random country variances.

The use of estimates of intra-unit correlations to determine the degree of clustering of child stunting or underweight is complicated by the fact that the total variance at community level is a function of mother’s HIV status. The estimates of intra-unit correlations from the variance components model (before the covariates are included in the model – not shown) suggest that only about five percent of the total variation in child undernutrition (4% for stunting; and 7% for wasting and underweight) in sub-Saharan Africa is attributable to country level factors, while more than 10 percent of the variation is attributable to community level factors (12% for stunting; 18% for wasting and 15% for underweight). Despite the country level variations in child undernutrition being partly explained by the GDP per capita, most of the variations at community/cluster level are attributable to unobserved factors rather than the explanatory factors included in this analysis. After controlling for the various covariates included in Table 3, less than five percent of the total unexplained variation in undernutrition is attributable to unobserved country level factors, while 10-15 percent of the total unexplained variation is attributable to unobserved community level factors. Although the effect of mother’s HIV status on stunting and underweight varies significantly at community level, this has minimal effect on the degree of clustering of these outcomes within communities, as the intra-community correlation coefficient for children whose mothers are infected with HIV is only one percentage point lower.

## Discussion and conclusions

Overall, the results presented above provide evidence of a higher risk of undernutrition among children whose mothers are infected with HIV, but there is no evidence of increased risk of undernutrition among paternal orphans, or among children in households where other adults are infected with HIV, or in communities or countries with higher HIV prevalence. Although the results from bivariate analyses show mixed patterns, the multivariate analysis results suggest that across countries and communities in sub-Saharan Africa, children aged under five whose mothers are infected with HIV have on average more than 25 percent higher odds of being stunted (28%), wasted (26%) or underweight (26%) compared to their counterparts of similar characteristics in households where no adult is infected with HIV. The fact that the vulnerability of children whose mothers are HIV positive becomes particularly apparent once socio-economic factors (i.e. urban/rural residence, household wealth status, and mother’s educational attainment) are controlled for suggests that bivariate associations are likely to conceal this association. Since the risk of HIV infection tends to be higher among the more affluent sub-groups of the population living in urban areas or in wealthier households (Magadi & Desta, 2009; Mishra et al., 2007), factors also associated with lower levels of malnutrition, failure to control for these factors is likely to influence the independent risk of parent’s HIV status on child malnutrition.

While some of the previous studies have suggested no significant difference in the prevalence of undernutrition in children by maternal HIV and survival status (Bridge et al., 2006; Nalwoga et al., 2010), others have suggested that maternal survival and HIV status are significant predictors of child health, including infant/child survival (Nakiyingi et al., 2003). Our findings relating to maternal HIV status are consistent with the latter which was based on a multivariate analysis controlling for the effect of other significant factors, unlike the former studies that were based on bivariate distributions. This supports the explanation given above, in the previous paragraph relating to bivariate and multivariate patterns.

The observed increased undernutrition among children whose mothers are HIV positive may be partly attributable to higher levels of malnutrition among children infected with HIV. Available evidence suggests that without intervention^2^, 20–45 percent of children born to HIV positive mothers would acquire the infection from their mothers through vertical transmission (WHO, 2010), and that the risk of undernutrition is significantly higher among children infected with HIV than among non infected children (Bunn, 2009; Nalwoga et al., 2010). Despite notable progress in recent years, mother-to-child transmission of HIV continues to account for a substantial portion of new HIV infections in many African Countries (UNAIDS & WHO, 2009), calling for intensified efforts to reduce the risk of mother-to-child transmission of HIV across the sub-Saharan Africa region. Nevertheless, Nalwoga et al. (2010) noted that the population-level impact of childhood HIV infection on nutritional status is unlikely to be extensive given the low HIV prevalence in children, and therefore, recommended that the response to undernutrition in children in Africa involves action on diverse fronts, including delivery of community-wide HIV and nutritional interventions as well as addressing the many interacting factors that contribute to childhood undernutrition.

Other possible mechanisms through which mother’s HIV infection may influence children’s nutritional status are reduced breastfeeding or lack of adequate parental care due to HIV/AIDS illness. The fact that children who were never breastfed have 2–4 times the odds of being malnourished compared to those who were breastfed for up to six months (Table 3), and that HIV positive mothers are significantly less likely to have breastfed their children (analysis not shown), suggests a possible indirect effect of mothers HIV status on child malnutrition through lack of breastfeeding. Nevertheless, the fact that the observed higher risk of undernutrition among children whose mothers are infected with HIV persists even after breastfeeding duration is controlled for suggests that other factors such as reduced parental care are possible mechanisms. The evidence of increased vulnerability for children whose mothers are infected with HIV calls for greater integration of child nutrition and HIV services for improved nutrition and survival chances of children in sub-Saharan Africa whose mothers are infected with HIV. Special attention should be on children who are particularly at an increased risk such as those whose mothers have no education or living in poverty.

It is interesting to note that the analysis presented here provides no evidence that paternal orphans or children in household where other adult household members (other than mother) are HIV positive, or living in communities or countries with higher HIV prevalence are more undernourished than those in less affected households or communities. These findings, though consistent with patterns observed in specific settings in sub-Saharan Africa (Bridge et al., 2006; Owen et al., 2009; Zidron et al., 2009), call for further research to better understand the possible mechanisms. Possible explanations for lack of differences in nutritional status between orphans and non-orphans have included the possibility that orphans live in wealthier households than non-orphans (Zidron, et al., 2009). However, our findings show no evidence of orphans being more vulnerable even after controlling for household wealth index, and therefore support the view that any nutrition interventions should be targeted at all vulnerable children, both orphaned and non-orphaned. The fact that paternal orphanhood or HIV status is not significant is an unexpected finding, especially since potential losses of income by the main salaried worker (the father is often the breadwinner), would be expected to impact adversely on children’s nutritional status.

It is important to note that even though overall there is no evidence of increased risk of malnutrition among children aged under five living in households where other adult household members, besides the mother, are HIV positive, the younger infants aged under one year in these households are significantly disadvantaged. The younger infants are generally less likely to be stunted or underweight than older children aged 1–4 years, but the risk of undernutrition (especially stunting and underweight) among younger infants in households where at least one adult is infected with HIV are significantly increased, relative to older children (Appendix iv). This may be partly attributable to reduced parental care having more adverse impact on younger infants, especially if the child’s mother has caring responsibilities for the HIV positive household member. The physical and emotional strain on the mother due to the caring demands is likely to affect, in particular, the younger children aged under one year for whom exclusive breastfeeding during the first six months plays a crucial role in nutritional status and overall well-being.

## Figures and Tables

**Table:** 

Name of variable	Measure
Outcome (dependent) variables	
Child stunted	Coded as: 1 = if the child has a height-for-age Z score less than −2 standard deviations relative to the WHO reference standard; and 0 = otherwise. This measures chronic undernutrition
Child wasted	Coded as: 1 = if the child has a height-for-weight Z score less than −2 standard deviations relative to the WHO reference standard; and 0 = otherwise. This measures acute undernutrition.
Child underweight	Coded as: 1 = if the child has a weight-for-age Z score less than −2 standard deviations relative to the WHO reference standard; and 0 = otherwise. This measures general undernutrition
Explanatory (independent) variables	
Parental survival and HIV status	
HIV status of adult household members (Ref = none positive)	Dummy variables for HIV status of adult household members, classified into three categories: none positive, mother positive, other adult household members positive.
Paternal orphan (Ref = no)	Coded as: 1 = if the child is a paternal orphan; 0 = otherwise
Child-level covariates	
Age of child (Ref = less than 1 year)	Dummy variables for age of child in completed years, classified into five categories: less than 1 year, 1 year, 2 years, 3 years, 4 years.
Sex of child (Ref = male)	Dichotomous variable for sex of child, coded as 1 if the child is female, and 0 if the child is male.
Birth order of child (Ref = fifth+)	Dummy variables for birth order of child, classified into five categories: first birth, second, third, fourth and fifth+.
Multiple births (Ref = singleton)	Dichotomous variable for type of birth, coded as: 1 for multiple or twin births, and 0 for singletons.
Birth interval (Ref = upto 24 months)	Dummy variables for preceding birth interval, classified into four categories: upto 24 months, 25–36 months, more than 36 months and first birth.
Duration of breastfeeding (Ref = never breastfed)	Dummy variables for duration of breastfeeding, classified into three categories: never breastfed, upto 6 months, more than 6 months.
Size of child at birth (Ref = small)	Dummy variables for reported size of child at birth, classified into three categories: small, average and large. This is used as a proxy for birth weight since data on birth weight is missing for a significant proportion of children born at home.
Mother and household covariates	
Mother’s age group (Ref = 15–19)	Dummy variables for five-year age groups of child’s mother, classified into five categories: 15–19, 20–24, 25–29, 30–34, 35+.
Mother’s marital status (ref = married)	Dichotomous variable for mother’s current marital status, coded as 1 if single; 0 if married/partnered.
Mother’s education level (Ref = none)	Dummy variables for mother’s highest educational attainment classified into three categories: none, primary, secondary.
Residence (Ref = urban)	Dichotomous variable for current place of residence, coded as 1 = if respondent was living in a rural area at the time of the survey; 0 = otherwise.
Wealth quintile (Ref: poorest)	DHS household wealth index^a^ derived from information on household possessions and amenities using Principal Components Analysis (PCA). The PCA scores are classified into wealth quintiles, the lowest quintile being the poorest.
Contextual variables	
Community/cluster level	
HIV prevalence	Percent of men and women of reproductive age in the community/cluster infected with HIV, derived from individual-level information. This is re-scaled so that one unit represents 10 percentage points.
Orphanhood prevalence	Proportion of children in the community/cluster who are orphans, derived from child-level information.
Community/cluster wealth index	Average wealth index of households in the community/cluster, derived from DHS household wealth index.
Country level	
HIV prevalence	Percent of men and women of reproductive age in the country infected with HIV, derived from individual-level information. This is re-scaled so that one unit represents 10 percentage points.
Orphanhood prevalence	Proportion of children in the country who are orphans, derived from child-level information.
Wealth	GDP per capita for country - estimates for year of survey obtained from the World Bank Development Indicators database^b^.

a Rutstein and Johnston (2004). *The DHS Wealth Index*. DHS Comparative Reports No.6. ORC Macro, Calverton, Maryland USA.

b Source: World Bank Development Indicators database – GDP estimates for respective survey years.http://data.worldbank.org/indicator/NY.GDP.PCAP.CD?page=1(last updated April 2011).

**Table:** 

Country	Stunted		Wasted		Underweight	
	Male	Female	Male	Female	Male	Female
Burkina Faso	39.5	35.3	18.4	17.2	39.4	36.2
Cameroon	31.5	29.8	4.8	5.3	17.1	19.0
DR Congo	39.2	33.7	9.9	7.8	29.9	26.6
Ethiopia	44.9	43.5	10.5	9.5	36.9	36.3
Ghana	30.4	25.1	6.5	6.9	20.9	20.2
Guinea	34.9	31.5	10.0	8.5	25.5	23.4
Kenya	32.7	25.9	6.3	3.6	22.3	16.8
Lesotho	35.5	32.0	4.2	3.3	17.3	18.5
Liberia	34.4	29.6	6.0	5.9	21.7	21.6
Malawi	49.3	44.4	4.4	4.4	21.2	22.1
Mali	32.4	31.0	14.1	11.5	30.2	29.4
Niger	51.3	46.6	11.3	9.5	44.5	42.3
Rwanda	45.6	43.9	4.3	3.6	22.7	22.2
Senegal	15.1	15.5	8.1	6.6	15.0	17.5
Sierra Leone	33.8	29.1	8.5	8.1	26.5	21.7
Swaziland	24.2	20.4	2.8	1.6	6.5	6.3
Zambia	40.5	35.0	5.2	3.8	19.6	16.9
Zimbabwe	28.0	24.8	5.5	5.1	15.1	14.9
Total	36.5	32.9	8.1	7.0	25.2	23.9

**Table:** 

Country	Child’s age in completed years					Total cases
	0.00	1.00	2.00	3.00	4.00	
Burkina Faso	12.5	44.9	45.0	46.8	39.2	2967
Cameroon	11.6	41.7	34.2	34.2	33.1	3241
DR Congo	13.3	33.9	38.7	44.9	51.9	3363
Ethiopia	17.0	51.2	50.2	51.5	53.0	4122
Ghana	11.0	33.3	30.9	32.5	31.2	3295
Guinea	9.3	38.1	44.0	40.8	40.4	2608
Kenya	12.4	40.2	37.1	29.9	29.1	2435
Lesotho	15.5	43.6	35.8	40.4	38.3	1480
Liberia	11.9	37.3	35.0	39.4	40.7	4216
Malawi	23.4	56.0	49.6	52.9	52.3	2269
Mali	10.5	46.0	39.2	35.4	30.3	3650
Niger	16.4	59.2	60.5	60.6	52.1	3708
Rwanda	16.8	55.0	50.5	52.1	51.4	3657
Senegal	6.0	19.7	16.0	20.4	16.4	2844
Sierra Leone	12.1	34.4	41.4	36.3	36.9	2060
Swaziland	10.0	35.9	27.3	17.5	20.4	1942
Zambia	17.1	49.1	40.7	40.2	42.0	4256
Zimbabwe	12.2	36.7	27.5	30.9	25.7	3636
Total	13.3	42.8	40.1	40.6	39.1	55749

**Table:** 

Country	Child’s age in completed years					Total cases
	0.00	1.00	2.00	3.00	4.00	
Burkina Faso	21.3	29.6	19.8	10.6	6.6	2967
Cameroon	4.1	9.8	5.7	1.9	2.9	3241
DR Congo	8.6	13.5	9.1	7.0	5.5	3363
Ethiopia	8.1	17.3	8.7	8.6	8.2	4122
Ghana	10.0	12.0	5.0	3.2	2.7	3295
Guinea	10.9	17.0	7.8	6.0	3.8	2608
Kenya	4.5	9.4	3.5	4.1	2.7	2435
Lesotho	5.1	6.1	2.6	2.3	2.0	1480
Liberia	7.1	11.5	5.1	2.9	2.3	4216
Malawi	4.7	6.6	4.2	3.2	2.5	2269
Mali	14.0	22.7	10.7	9.1	6.7	3650
Niger	9.8	18.8	10.4	6.6	6.1	3708
Rwanda	4.1	8.8	3.1	1.3	1.9	3657
Senegal	6.0	13.0	6.7	4.5	6.2	2844
Sierra Leone	8.2	10.4	6.9	9.7	5.6	2060
Swaziland	2.7	4.2	0.5	1.7	1.4	1942
Zambia	5.8	6.6	4.4	2.1	2.8	4256
Zimbabwe	4.7	7.5	6.3	4.1	3.8	3636
Total	8.0	12.9	7.0	5.1	4.3	55749

**Table:** 

Country	Child’s age in completed years					Total cases
	0.00	1.00	2.00	3.00	4.00	
Burkina Faso	21.5	52.1	49.1	37.5	29.2	2967
Cameroon	8.8	29.0	20.3	15.5	17.2	3241
DR Congo	12.0	32.8	32.5	29.4	34.9	3363
Ethiopia	14.6	46.4	41.5	41.2	41.5	4122
Ghana	12.6	28.9	24.5	20.3	15.6	3295
Guinea	10.8	33.3	33.9	25.1	23.5	2608
Kenya	8.4	28.0	23.9	20.5	18.4	2435
Lesotho	5.0	20.7	24.1	18.5	24.6	1480
Liberia	13.2	30.4	25.8	19.6	19.4	4216
Malawi	13.6	28.1	26.5	19.4	19.8	2269
Mali	14.4	45.4	38.6	29.3	23.4	3650
Niger	18.5	57.9	53.5	49.0	40.1	3708
Rwanda	11.4	35.7	27.0	17.9	18.7	3657
Senegal	7.0	21.6	19.8	17.4	17.1	2844
Sierra Leone	10.1	29.7	27.4	26.4	29.0	2060
Swaziland	3.4	11.6	7.2	4.0	5.0	1942
Zambia	8.3	28.5	20.7	16.5	16.5	4256
Zimbabwe	6.9	20.4	19.4	15.8	13.0	3636
Total	11.6	33.6	29.9	25.1	23.5	55749

**Table:** 

Country	Non orphan		Paternal orphan	
	Percent	Cases	Percent	Cases
Burkina Faso	37.5	2897	37.7	56
Cameroon	30.9	3133	23.4	80
DR Congo	36.3	3247	34.2	83
Ethiopia	44.1	4005	49.5	104
Ghana	27.8	3197	28.0	58
Guinea	33.4	2525	33.9	56
Kenya	29.6	2314	20.8	73
Lesotho	32.8	1298	39.1	138
Liberia	32.8	4062	39.1	81
Malawi	46.4	2181	52.5	69
Mali	31.9	3549	39.7	46
Niger	49.3	3636	36.0	53
Rwanda	44.7	3537	49.5	101
Senegal	15.4	2755	13.6	66
Sierra Leone∗∗	32.2	1969	16.9	59
Swaziland	22.0	1861	26.7	71
Zambia	37.8	4149	36.5	86
Zimbabwe	26.1	3423	31.5	189

∗∗Chi-Square *p* < 0.01.

**Table:** 

Country	Non orphan		Paternal orphan	
	Percent	Cases	Percent	Cases
Burkina Faso	18.0	2897	12.9	56
Cameroon	5.1	3133	6.5	80
DR Congo	8.9	3247	4.2	83
Ethiopia	10.1	4005	9.1	104
Ghana	6.8	3197	2.0	58
Guinea	9.5	2525	1.8	56
Kenya	4.8	2314	5.2	73
Lesotho	3.8	1298	3.8	138
Liberia	5.9	4062	4.5	81
Malawi	4.6	2181	1.7	69
Mali∗∗	13.1	3549	1.7	46
Niger	10.4	3636	14.0	53
Rwanda	4.0	3537	2.8	101
Senegal	7.2	2755	12.3	66
Sierra Leone	8.3	1969	7.0	59
Swaziland	2.0	1861	5.4	71
Zambia∗∗	4.3	4149	14.1	86
Zimbabwe∗	5.1	3423	8.6	189

∗Chi–Square *p* < 0.05; ∗∗Chi–Square *p* < 0.01.

**Table:** 

Country	Non orphan		Paternal orphan	
	Cases		Cases	
Burkina Faso	38.0	2897	31.1	56
Cameroon	18.2	3133	15.6	80
DR Congo	28.3	3247	23.3	83
Ethiopia	36.7	4005	35.7	104
Ghana	20.8	3197	14.0	58
Guinea	24.7	2525	23.2	56
Kenya	19.5	2314	21.8	73
Lesotho	18.0	1298	15.8	138
Liberia	21.8	4062	21.3	81
Malawi	21.7	2181	21.3	69
Mali	29.7	3549	41.4	46
Niger	43.6	3636	42.0	53
Rwanda	22.6	3537	17.9	101
Senegal	16.2	2755	20.0	66
Sierra Leone	24.4	1969	18.3	59
Swaziland	6.4	1861	5.4	71
Zambia	18.2	4149	25.0	86
Zimbabwe∗	14.6	3423	20.5	189

∗Chi–Square *p* < 0.05.

**Table:** 

Parameter	Stunted	Wasted	Underweight
*Fixed Effects*			
Constant	−0.87(0.141)	−1.61(0.226)	−0.52(0.146)
Hhold HIV Status (none +)			
Mother HIV+	0.54(0.126)∗	0.34 (0.193)	0.39(0.158)∗
Other adults HIV+	0.32(0.141)∗	0.38(0.184)∗	0.45(0.158)∗
Paternal orphan (non-orphan)			
Paternal orphan	−0.13(0.067)∗	−0.11(0.132)	−0.21(0.077)∗
Age of child (<1 year)			
1 year	1.30 (0.043)∗	0.19(0.058)∗	0.81(0.044)∗
2 years	1.22(0.044)∗	−0.57(0.066)∗	0.63(0.045)∗
3 years	1.26(0.044)∗	−0.94(0.071)∗	0.34(0.046)∗
4 years	1.23(0.045)∗	−1.06(0.076)∗	0.29(0.048)∗
Mum HIV+ (<1 year, none+)			
1 year	0.03(0.151)	−0.07(0.239)	−0.08(0.185)
2 years	−0.34(0.155)∗	−0.18(0.287)	−0.19(0.190)
3 years	−0.31(0.151)∗	−0.10(0.300)	−0.11(0.189)
4 years	−0.73(0.157)∗	−0.37(0.354)	−0.39(0.201)
Others in HH+ (<1 year, none+)			
1 year	−0.22(0.175)	−0.47(0.261)	−0.53(0.200)∗
2 years	−0.36(0.179)∗	−0.40(0.313)	−0.47(0.206)∗
3 years	−0.57(0.189)∗	−0.56(0.384)	−0.62(0.225)∗
4 years	−0.56(0.192)∗	−0.83(0.458)	−0.76(0.237)∗
*Contextual factors*			
HIV prevalence in community	−0.03(0.012)∗	−0.04(0.025)	−0.01(0.014)
HIV prevalence in country	0.19(0.111)	−0.28(0.133)∗	−0.12(0.099)
GDP per capita - country	−0.68(0.235)∗	−0.23(0.282)	−0.66(0.211)∗
*Random effects*			
Cluster – constant	0.28(0.016)∗	0.44(0.040)∗	0.23(0.017)∗
Cluster – constant/mum HIV+	−0.08(0.048)	–	−0.18(0.065)∗
Cluster – Mum HIV+	0.17(0.120)	–	0.39(0.180)∗
Country – constant	0.11(0.038)∗	0.14(0.051)∗	0.09(0.030)∗

∗Statistical significance at 5% level – *p* < 0.05. Covariates controlled for: sex of child, birth order, multiple/twin birth, preceding birth interval, breastfeeding, size of child at birth, urban/rural residence, mother’s education, mother’s age group, single parenthood, and household wealth index.

**Table 1:** Distribution of analysis sample size by age of child.

Country	Childs age in completed years					Total cases
	0.00	1.00	2.00	3.00	4.00	
Burkina Faso 2003	620	585	584	628	550	2967
Cameroon 2004	725	721	623	584	588	3241
DR Congo 2007	711	693	686	641	632	3363
Ethiopia 2005	846	784	781	863	848	4122
Ghana 2003	672	709	652	689	573	3295
Guinea 2005	605	527	512	451	513	2608
Kenya 2003	547	479	472	521	416	2435
Lesotho 2004/5	365	293	289	269	264	1480
Liberia 2007	962	836	878	851	689	4216
Malawi 2004	488	540	407	424	410	2269
Mali 2006	822	737	711	711	669	3650
Niger 2006	809	756	752	759	632	3708
Rwanda 2005	778	762	835	632	650	3657
Senegal 2005	677	625	522	541	479	2844
Sierra Leone 2008	461	459	396	404	340	2060
Swaziland 2006	434	441	370	347	350	1942
Zambia 2007	893	940	844	810	769	4256
Zimbabwe 2005/6	804	757	687	671	717	3636
Total	12219	11644	11001	10796	10089	55749

Note: Cases presented here are fewer than reported in DHS reports since the analysis is restricted to children in households samples for HIV testing.

**Table 2:** (a) Percent of children aged 0–4 years stunted in each country by household HIV status. (b) Percent of children aged 0–4 years wasted in each country by household HIV status. (c) Percent of children aged 0–4 years underweight by household HIV status.

Country	None in HH HIV positive		Mother HIV positive		Others in HH positive	
	Percent	Cases	Percent	Cases	Percent	Cases
(a)						
Burkina Faso	37.6	2832	45.2	38	27.6	97
Cameroon	31.2	2892	27.2	163	24.0	186
DR Congo∗	36.7	3267	29.0	38	19.6	58
Ethiopia∗	44.3	4010	51.7	65	26.2	47
Ghana	27.9	3196	24.1	57	24.2	42
Guinea	32.9	2526	45.8	24	41.4	58
Kenya	29.7	2202	27.4	152	20.5	81
Lesotho∗∗	31.7	996	42.5	327	30.3	159
Liberia	32.3	4070	27.7	62	23.2	84
Malawi∗	47.5	1936	46.2	243	33.3	90
Mali	31.8	3560	27.8	40	24.5	50
Niger	49.2	3639	36.8	23	32.3	46
Rwanda	45.2	3449	35.8	144	38.2	64
Senegal	15.1	2770	25.0	19	25.0	55
Sierra Leone	31.7	2015	19.2	24	16.7	21
Swaziland	20.2	925	25.4	627	22.4	390
Zambia∗	38.5	3398	35.6	501	32.0	357
Zimbabwe∗∗	25.4	2569	31.0	703	24.7	364

(b)						
Burkina Faso	17.7	2832	22.6	38	18.7	97
Cameroon	5.1	2892	7.0	163	3.4	186
DR Congo	8.9	3267	6.5	38	3.6	58
Ethiopia	10.1	4010	8.3	65	7.0	47
Ghana∗	6.6	3196	5.6	57	18.2	42
Guinea∗	9.4	2526	20.8	24	1.7	58
Kenya	5.0	2202	4.8	152	2.3	81
Lesotho	3.8	996	3.4	327	4.1	159
Liberia	5.8	4070	6.4	62	13.0	84
Malawi	4.6	1936	3.3	243	3.6	90
Mali	12.7	3560	22.2	40	12.5	50
Niger	10.4	3639	20.0	23	12.9	46
Rwanda	4.1	3449	2.5	144	1.8	64
Senegal	7.4	2770	12.5	19	4.1	55
Sierra Leone	8.3	2015	15.4	24	5.6	21
Swaziland	1.7	925	2.8	627	2.0	390
Zambia	4.4	3398	5.1	501	4.3	357
Zimbabwe	5.3	2569	5.9	703	4.4	364

(c)						
Burkina Faso	37.8	2832	45.2	38	36.8	97
Cameroon∗	18.7	2892	12.0	163	13.5	186
DR Congo∗∗	28.7	3267	22.6	38	5.4	58
Ethiopia	36.8	4010	30.5	65	31.0	47
Ghana	20.7	3196	14.8	57	21.2	42
Guinea	24.3	2526	33.3	24	25.9	58
Kenya∗	19.8	2202	20.8	152	9.1	81
Lesotho	17.7	996	19.5	327	15.2	159
Liberia	21.7	4070	17.0	62	20.3	84
Malawi	21.5	1936	22.9	243	21.4	90
Mali	29.7	3560	30.6	40	32.7	50
Niger	43.5	3639	36.8	23	33.3	46
Rwanda∗	22.8	3449	13.3	144	18.5	64
Senegal	16.1	2770	25.0	19	18.8	55
Sierra Leone	24.2	2015	11.5	24	22.2	21
Swaziland	5.3	925	8.2	627	6.0	390
Zambia	18.6	3398	18.3	501	14.9	357
Zimbabwe∗∗	14.0	2569	19.7	703	13.2	364

∗Chi–Square *p* < 0.05, ∗∗Chi–Square *p* < 0.01.

**Table 3:** Multilevel logistic regression average odds ratios and random parameter estimates for child malnutrition in SSA (95% confidence intervals given in square brackets).

Parameter	Stunted	Wasted	Underweight
*Individual child/family factors*			
Hhold HIV Status (none +)			
Mother HIV+	1.28[1.16–1.42]∗	1.26[1.02–1.55]∗	1.26[1.11–1.43]∗
Other adults HIV+	0.98[0.87–1.09]	1.03[0.83–1.27]	0.97[0.85–1.11]
Paternal orphan (non-orphan)			
Paternal orphan	0.86[0.75–0.98]∗	0.89[0.69–1.15]	0.80[0.69–0.93]∗
Age of child (<1 year)			
1 year	3.65[3.36–3.96]∗	1.18[1.06–1.32]∗	2.18[2.01–2.38]∗
2 years	3.26[3.00–3.54]∗	0.55[0.49–0.63]∗	1.82[1.67–1.98]∗
3 years	3.39[3.12–3.68]∗	0.38[0.33–0.44]∗	1.37[1.25–1.49]∗
4 years	3.21[2.95–3.49]∗	0.33[0.29–0.38]∗	1.28[1.17–1.40]∗
Sex of child (male)			
female	0.81[0.78–0.84]∗	0.82[0.76–0.88]∗	0.88[0.84–0.92]∗
Multiple/twin (no)			
yes	2.13[1.89–2.39]∗	1.23[1.01–1.49]∗	2.03[1.80–2.30]∗
Birth order of child (fifth+)			
second	0.82[0.76–0.89]∗	0.87[0.76–1.00]	0.81[0.74–0.88]∗
third	0.91[0.84–0.98]∗	0.87[0.76–1.00]	0.89[0.82–0.96]∗
fourth	0.92[0.86–0.99]∗	0.94[0.83–1.07]	0.97[0.89–1.04]
First birth (fifth + birth, with <=24 months preceding interval)	0.65[0.59–0.72]∗	0.92[0.77–1.09]	0.69[0.62–0.77]∗
Birth interval (<= 24 months)			
25–36 months	0.86[0.81–0.91]∗	0.95[0.85–1.06]	0.89[0.83–0.95]∗
More than 36 months	0.74[0.70–0.79]∗	0.98[0.88–1.10]	0.75[0.71–0.81]∗
Child breastfed (never)			
Upto 6 months	0.47[0.39–0.56]∗	0.44[0.32–0.61]∗	0.22[0.18–0.27]∗
More than 6 months	1.18[1.01–1.38]∗	0.97[0.72–1.31]	1.13[0.95–1.34]
Size of child at birth (small)			
Average	0.74[0.70–0.78]∗	0.76[0.70–0.84]∗	0.63[0.60–0.67]∗
Larger than average	0.62[0.59–0.66]∗	0.59[0.53–0.65]∗	0.46[0.43–0.49]∗
Residence (urban)			
rural	1.32(1.23–1.42)∗	0.88[0.78–0.99]∗	1.16[1.07–1.25]∗
Mother’s education (none)			
primary	0.86[0.81–0.91]∗	0.88[0.79–0.97]∗	0.78[0.74–0.83]∗
secondary +	0.67[0.62–0.73]∗	0.76[0.66–0.87]∗	0.58[0.53–0.63∗
Mother’s age (15–19)			
20–24	0.85[0.77–0.94]∗	1.17[0.99–1.37]	0.91[0.81–1.01]
25–29	0.78[0.70–0.87]∗	1.07[0.89–1.29]	0.84[0.74–0.95]∗
30–34	0.73[0.65–0.83]∗	1.11[0.91–1.36]	0.79[0.70–0.91]∗
35+	0.69[0.60–0.78]∗	1.12[0.90–1.39]	0.78[0.68–0.90]∗
Single parent (no)			
yes	1.15[1.06–1.23]∗	1.09[0.95–1.25]	1.24[1.15–1.35]∗
Wealth index (poorest)			
poorer	0.93[0.87–0.98]∗	0.97[0.87–1.07]	0.90[0.84–0.96]∗
middle	0.88[0.83–0.94]∗	0.87[0.78–0.97]∗	0.85[0.79–0.91])∗
richer	0.78[0.73–0.84]∗	0.82[0.72–0.92]∗	0.74[0.69–0.80]∗
richest	0.53[0.48–0.58]∗	0.65[0.55–0.76]∗	0.50[0.45–0.55]∗
*Contextual factors*			
HIV prevalence in community	0.97[0.95–0.99]∗	0.97[0.92–1.01]	0.99[0.96–1.02]
HIV prevalence in country	1.21[0.97–1.50]	0.76[0.58–0.98]∗	0.89[0.73–1.08]
GDP per capita – Country	0.51[0.32–0.80]∗	0.80[0.46–1.39]	0.52[0.34–0.78]∗
*Random variance*			
*Cluster/Community level*^a^			
Intercept	0.28[0.25, 0.31]∗	0.44[0.36, 0.52]∗	0.23[0.20, 0.26]∗
Intercept/mum HIV+	−0.07[-0.17, 0.03]	–	−0.17[-0.30,-0.05]∗
Mum HIV+	0.18[-0.06, 0.42]	–	0.36[0.01, 0.71]∗
*Country level*			
Intercept	0.11[0.04, 0.18]∗	0.14[0.04, 0.24]∗	0.09[0.03, 0.15]∗

∗Statistical significance at 5% level – *p* < 0.05.

a The total variance at community/cluster level is the variance of the sum of the two random variables for INTERCEPT and MUM HIV+, and is given by σ^2^_v0_ +2σ_v0v1_W_ijk_ + σ^2^_v1_W^2^_ijk_, where: σ^2^_v0_ is the community level variance for INTERCEPT (0.28 for stunting and 0.23 for underweight); σ^2^_v1_ is the community level variance for MUM HIV+ (0.18 for stunting and 0.36 for underweight); σv0v1 is the covariance between INTERCEPT and MUM HIV+(−0.07 for stunting and −0.17 for underweight); and W_ijk_takes the value 1 if the mother is infected with HIV,and a value of 0 otherwise.

## Footnotes

1 Source: World Bank Development Indicators database – GDP estimates for respective survey years.http://data.worldbank.org/indicator/NY.GDP.PCAP.CD?page=1(last updated April 2011).

2 Only 6–28% of pregnant women in sub-Saharan Africa were tested for HIV during 2004–2008, and the proportion of HIV positive pregnant women in sub-Saharan Africa who received antiretrovirals for preventing mother-to-child transmission of HIV ranges from 9% in 2004 to 45% in 2008 (WHO, UNIAIDS, & UNICEF, 2009:98,99).
